# Engineering of Biosynthesis Pathway and NADPH Supply for Improved L-5-Methyltetrahydrofolate Production by *Lactococcus lactis*

**DOI:** 10.4014/jmb.1910.10069

**Published:** 2019-12-24

**Authors:** Chuanchuan Lu, Yanfeng Liu, Jianghua Li, Long Liu, Guocheng Du

**Affiliations:** 1 Key Laboratory of Carbohydrate Chemistry and Biotechnology, Ministry of Education, Jiangnan University, Wuxi 2422, P.R. China; 2 Key Laboratory of Industrial Biotechnology, Ministry of Education, Jiangnan University, Wuxi 141, P.R. China

**Keywords:** L-5-Methyltetrahydrofolate, *Lactococcus lactis*, metabolic engineering, strengthening synthetic pathway, NADPH supply

## Abstract

L-5-methyltetrahydrofolate (5-MTHF) is one of the biological active forms of folate, which is widely used as a nutraceutical. However, low yield and serious pollution associated with the chemical synthesis of 5-MTHF hampers its sustainable supply. In this study, 5-MTHF production was improved by engineering the 5-MTHF biosynthesis pathway and NADPH supply in *Lactococcus lactis* for developing a green and sustainable biosynthesis approach. Specifically, overexpressing the key rate-limiting enzyme methylenetetrahydrofolate reductase led to intracellular 5-MTHF accumulation, reaching 18 μg/l. Next, 5-MTHF synthesis was further enhanced by combinatorial overexpression of 5-MTHF synthesis pathway enzymes with methylenetetrahydrofolate reductase, resulting in 1.7-fold enhancement. The folate supply pathway was strengthened by expressing *folE* encoding GTP cyclohydrolase I, which increased 5-MTHF production 2.4-fold to 72 μg/l. Furthermore, glucose-6-phosphate dehydrogenase was overexpressed to improve the redox cofactor NADPH supply for 5-MTHF biosynthesis, which led to a 60% increase in intracellular NADPH and a 35% increase in 5-MTHF production (97 μg/l). To reduce formation of the by-product 5-formyltetrahydrofolate, overexpression of 5-formyltetrahydrofolate cyclo-ligase converted 5-formyltetrahydrofolate to 5,10-methyltetrahydrofolate, which enhanced the 5-MTHF titer to 132 μg/l. Finally, combinatorial addition of folate precursors to the fermentation medium boosted 5-MTHF production, reaching 300 μg/l. To the best of our knowledge, this titer is the highest achieved by *L. lactis*. This study lays the foundation for further engineering of *L. lactis* for efficient 5-MTHF biosynthesis.

## Introduction

L-5-methyltetrahydrofolate (5-MTHF) is one of the reduced forms of folate. 5-MTHF has important biologically active roles as a key one-carbon donor, and is also the main form of folate in plasma, either circulating in free form or bound to plasma proteins [1]. 5-MTHF can be directly absorbed and utilized in the circulation without additional metabolic steps and has better absorption than synthetic folic acid. The latter is non-reduced and methylated, and needs to be first reduced to 7,8-dihydrofolate (DHF) by dihydrofolate reductase (DHFR), and further reduced and methylated to 5,6,7,8-tetrahydrofolate (THF) and 5-MTHF, respectively [2, 3]. 5-MTHF acts as a methyl donor during the synthesis of methionine from homocysteine in the human body [4]. The lack of 5-MTHF leads to a reduction in the one-carbon unit required for the methylation reaction, which in turn leads to the accumulation of homocysteine, which is associated with human vascular disease, neural tube defects, and Alzheimer's disease [5-7]. 5-MTHF is the only form of folate that can cross the blood-brain barrier, which helps prevent fetal neural tube defects, arteriosclerosis, and other health problems [8, 9]. Humans and animals cannot synthesize folate due to the lack of the corresponding folate synthesis genes. Therefore, folate must be acquired in the diet or by absorption from intestinal bacteria [10, 11]. However, the folate content is low in natural foods and considerable loss of folate occurs during cooking. Insufficient folate intake is a common global problem [12]. To prevent folate deficiency, some countries allow the supplementation of foods with synthetic folic acid [13, 14]. However, using a stable isotope tracer, Wright et al. found that the absorption of natural folate and synthetic folic acid by the human body is different [15]. Moreover, whether supplementation with synthetic folic acid poses health risks is a contentious issue [16-18]. Therefore, supplementation with natural reduced folate, especially biologically active reduced 5-MTHF that has been reduced and methylated prior to absorption, is a more favorable option, since there would be no side effects over a broad intake range and more efficient bioavailability [2].

The production of 5-MTHF via chemical synthesis mainly uses folic acid as a precursor. However, this chemical synthesis is a complex process that requires a lot of energy and generates serious pollution. As well, the chemical synthesis generates a large amount of (6R, S)-5-methyltetrahydrofolate ((6R, S)-5-MTHF) that is the racemate of L-5-MTHF and has no biological activity [19]. Therefore, 5-MTHF must be separated from (6R, S)-5-MTHF to obtain biologically active 5-MTHF. This separation reduces the recovery rate considerably and results in high cost 5-MTHF production. Efficient and cost-effective production of 5-MTHF is desirable.

**Fig. 1:**
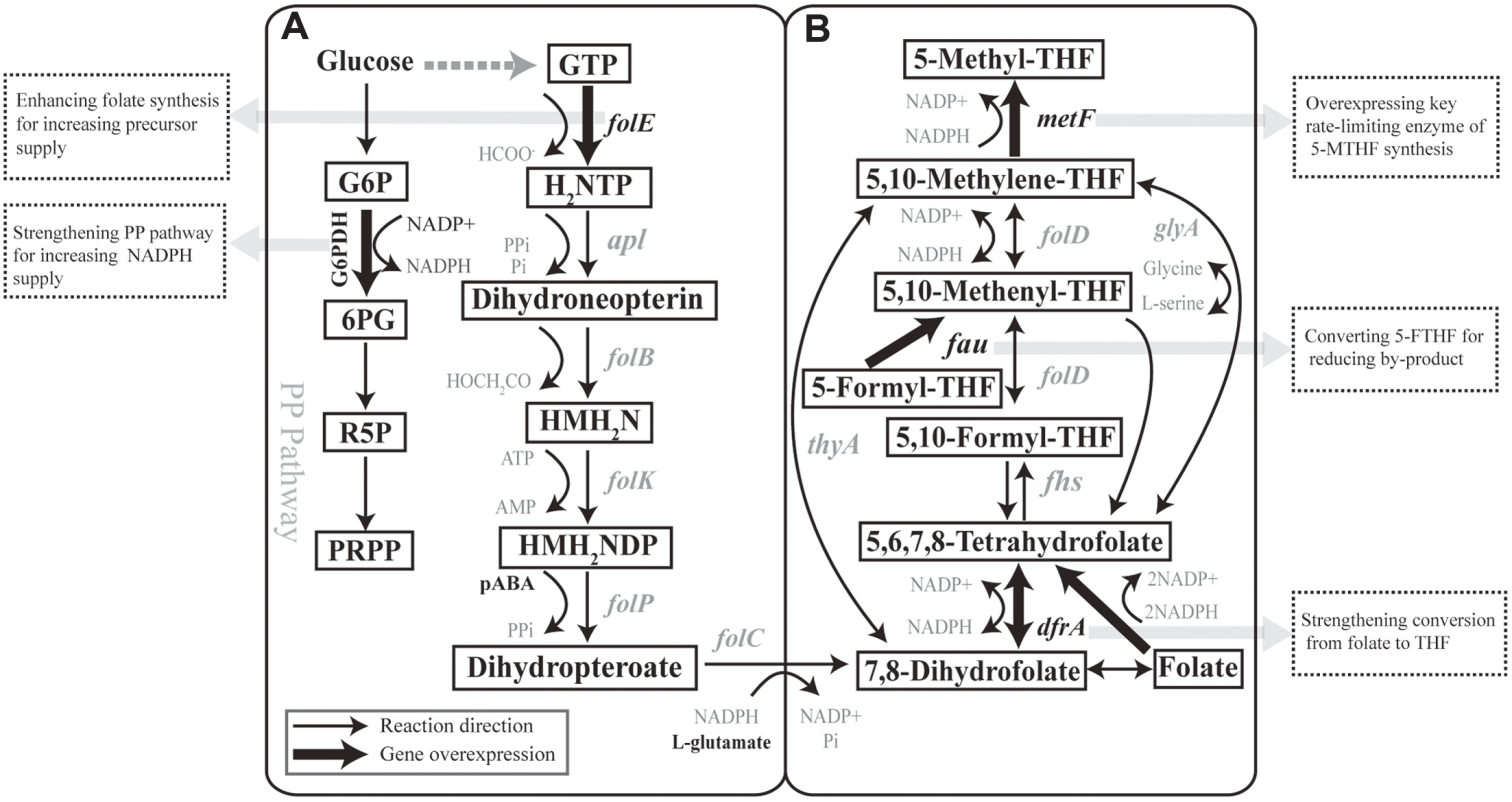
Folate metabolic pathway in *L. lactis* NZ9000 and engineering strategies with target genes. (**A**) Biosynthetic pathway of folates and pentose phosphate pathway. H_2_NTP, 7,8-Dihydroneopterin 3'-triphosphate; HMH_2_N, 6- Hydroxymethyl-7,8-dihydropterin; HMH_2_NDP, 6-Hydroxymethyl-7,8-dihydropterin diphosphate; *p*ABA, *para*-aminobenzoic acid; G6P, glucose 6-phosphate; 6PG, 6-phosphogluconate; P5P, Ribulose 5-phosphate; PRPP, Phosphoribosyl diphosphate. (**B**) Biosynthetic pathway of 5-MTHF from 7,8-dihydrofolate. Bold black arrows represent genes involved in folate metabolism that are overexpressed in engineered strains.

Many forms of folate can be produced by microorganisms, which provide a promising environmentally friendly and economically competitive approach compared to multi-stage chemical processes [20]. Engineering of the folate synthesis pathway for improved total folate production has been carried out. However, pathway engineering focusing on 5-MTHF production has not been systematically investigated [21-23]. *L. lactis* holds generally regarded as safe (GRAS) status and is one of the most commonly used lactic acid bacteria in fermented dairy products, fermented meat products, and fermented vegetables, which is favorable for producing 5-MTHF [24]. In addition, *L. lactis* is the most extensively studied lactic acid bacterium and has clear genetic background and efficient genetic engineering tools. Lastly, many previous studies about folate biosynthesis have mainly focused on *L. lactis*, which confirmed that *L. lactis* has strong folate biosynthetic ability and is likely to be engineered as a highly efficient host for 5-MTHF biosynthesis [21]. Therefore, the production of 5-MTHF by *L. lactis* fermentation could have unique advantages and a wider range of applications [25]. The biosynthesis of folate consists of a series of pathways, including the pterin, *p*-aminobenzoic acid (*p*ABA), and glutamate pathways [21, 26, 27]. Folate is formed using pterin, *p*ABA, and glutamic acid as direct precursors and is catalyzed by dihydrofolate synthase (Fig. 1A). DHF is reduced to THF with the participation of NADPH catalyzed by DHFR. THF is further methylated and then converted to 5-MTHF (Fig. 1B).

The main purpose of this study was to improve 5-MTHF production by *L. lactis* NZ9000 through metabolic engineering. Strengthening the 5-MTHF synthesis pathway from folate was first carried out to realize 5-MTHF accumulation. Next, the precursor supply pathways of folate were engineered to increase metabolic flux and lead to the overproduction of folate. Then, the intracellular NADPH level was elevated to facilitate 5-MTHF production by providing reducing power for the reduction reactions from folate to 5-MTHF. Finally, reducing the formation of the 5-formyltetrahydrofolate (5-FTHF) by-product and optimizing key precursor addition were carried out to further promote 5-MTHF synthesis. The developed engineering strategies and 5-MTHF-producing strain should be a good starting point for efficient 5-MTHF bioproduction in *L. lactis*.

## Materials and Methods

### Microorganisms and Cultivation Conditions

*L. lactis* NZ9000 and its derivatives were used. *Escherichia coli* JM109 was used for cloning purposes. Plasmids pMG36e and pTD6 were used to express various genes in *L. lactis*. The constructed strains are summarized in Table 1. *L. lactis* NZ9000 and its derivatives were grown at 30°C without agitation in M17 broth medium (Hope Biotechnology, Qingdao, China) supplemented with 5 g/L glucose (GM17). *E. coli* JM109 was cultured at 37°C in Luria-Bertani broth and on Luria-Bertani agar. When required, antibiotics were added in the medium. Erythromycin was added at 200 mg/l for *E. coli* and 5 mg/l for *L. lactis*. Tetracycline was added at 10 mg/l for *E. coli* and 5 mg/ml for *L. lactis*.

Fermentation experiments using *L. lactis* for 5-MTHF production were carried out. Seed culture was cultivated in 10 ml Eppendorf tubes for 10 h (late exponential phase) and 1% of the culture was added to a 50 ml shake flask with 40 ml of GM17 medium [28] and the temperature was maintained at 30°C. For experiments to test the effects of key precursor addition on 5-MTHF production, 50 mg/l *p*ABA, 300 mg/l glutamate, and 30 mg/l guanosine triphosphate (GTP) were added respectively or in combination to the fermentation medium.

### DNA Manipulation

All manipulations were performed according to a standard protocol [29]. PrimeSTAR Max DNA Polymerase (TaKaRa Bio, China) was used for PCR applications. Genomic DNA of *L. lactis* was isolated using the TIANamp Bacteria DNA Kit (Tiangen Biotech, China). The plasmid construction method utilized the ClonExpress II One-Step Cloning Kit (Vazyme, China). The transfer of plasmid into *L. lactis* was done as previously described [30], and *L. lactis* were made electrocompetent by growing in GM17 medium containing 2.5% glycine and 170 g/l sucrose [30].

### Overexpression of Genes in 5-MTHF Synthesis Pathway

The primers used are listed in Supplementary Table S1. The genes in the 5-MTHF synthesis pathway, including *metF* encoding MTHFR, *dfrA* encoding DHFR, *folD* encoding methylenetetrahydrofolate dehydrogenase, *glyA* encoding glycine hydroxymethyltransferase, and *thyA* encoding thymidylate synthase were amplified by PCR using *L. lactis* genomic DNA as template. The genes were inserted separately into plasmid pMG36e downstream of the P_32_ promoter using the ClonExpress II One-Step Cloning Kit, generating the corresponding overexpression plasmids. The plasmid construction process is depicted in Table 1. The plasmids were transformed into *L. lactis* NZ9000 by electrotransformation, yielding strains *L. lactis* M, *L. lactis* A, *L. lactis* D, *L. lactis* g, and *L. lactis* t.

To further enhance 5-MTHF synthesis, *drfA*, *glyA*, and *thyA* genes were amplified from genomic DNA by PCR.

These genes were respectively cloned into plasmid pMG36e-M downstream of *metF* gene, using the method described previously. Plasmids pMG36e-MA, pMG36e-Mg, and pMG36e-Mt were transformed into *L. lactis* NZ9000, yielding strains *L. lactis* MA, *L. lactis* Mg, and *L. lactis* Mt, respectively.

### Gene Overexpression to Strengthen Folate Synthetic Pathway

GTP cyclohydrolase I is one of the key rate-limiting enzymes in folate biosynthesis [21, 31]. To enhance folate synthesis, *folE* was amplified from genomic DNA and cloned into the pMG36e-MA and pMG36e-Mg plasmids to obtain pMG36e-MAE and pMG36e-MgE, respectively. The latter were transformed into *L. lactis* NZ9000 to obtain the *L. lactis* MAE and *L. lactis* MgA strains.

Overexpression of G6PDH allows more carbon flux to flow to the pentose phosphate pathway, which accelerates intracellular NADPH reduction (Fig. 1A) [32]. The G6PDH gene was amplified from *L. lactis* NZ9000 genomic DNA and was cloned into plasmid pTD6, generating the plasmid pTD6-G. It was transformed into *L. lactis* MAE, yielding the strains *L. lactis* MAE-G.

5-formyltetrahydrofolate cyclo-ligase coding by gene *fau* catalyzes the conversion of 5-FTHF to 5,10-methenyltetrahydrofolate, which reverts the folate metabolic flow back to the 5-MTHF synthesis pathway, thus facilitating the synthesis of 5-MTHF. The fau gene was cloned from the genome and cloned into the plasmid pTD6-G to obtain the plasmid pTD6-Gf. It was transformed into *L. lactis* MAE, yielding the strains *L. lactis* MAE-Gf.

### Extraction of Folates

*L. lactis* cells were harvested from the fermentation medium by centrifugation at 6,000 ×*g* for 5 min and resuspended in phosphate buffer (50 mM KH_2_PO_4_, pH 7.2), containing 1% ascorbic acid and 0.1% β-mercaptoethanol to prevent 5-MTHF from being oxidized. The sample was at 37°C for 1 h in the presence of 5 mg/ml lysozyme (Sangon Biotech, China) to digest the cell wall. This was followed by incubation at 100°C for 10 min to dissolve the cells and folate was released from folate-binding proteins. Cell debris was removed by centrifugation at 10,000 ×*g* for 5 min. Rat serum (3% v/v) was added to the sample as a source of γ-glutamyl hydrolase and incubated at 37°C for 3 h to deconjugate the glutamate tail. The serum was inactivated and the protein was precipitated by incubation at 100°C for 5 min and the precipitate was removed by centrifugation.

### Determination of 5-MTHF by High Performance Liquid Chromatography (HPLC)

5-MTHF was analyzed by HPLC using the 1260 series apparatus (Agilent, USA) and an ODS-2 Hypersil column (350 × 4.6 mm) with fluorescence detector (295 nm/356 nm) [33]. The mobile phase was 50 mM KH_2_PO_4_ buffer (pH 3.0) containing 7.5% acetonitrile, which was applied at a flow rate of 1 ml/min. The retention time of 5-MTHF was 7.2 min. The concentrations of the 5-MTHF standard were 2, 1, 0.75, 0.5, 0.2, and 0.1 mg/l. The linear equation describing the standard curves for 5-MTHF was y = 0.3558x + 2.7375 (R^2^ = 0.9991).

### Determination of Folate Derivatives by Ultraperformance Liquid Chromatography-Tandem Mass Spectrometry (UHPLC–MS/MS)

Folic acid, THF, 5-FTHF, and 5-MTHF were determined by UHPLC–MS/MS. The standard stock solution of folic acid was dissolved to 2 g/l with 0.5 N NH_3_·H_2_O and then diluted using extraction buffer to a gradient of concentrations. The standard displayed good linearity in the concentration range of 0.4 to 4 mg/l.

Analysis of the folates was performed on a UHPLC–MS/MS system consisting of an Acquity UHPLC coupled to a Xevo-TQS triple quadrupole mass spectrometer equipped with an electrospray ionization probe (Waters Corporation, USA). Chromatographic separation was completed using an Atlantis dC18 reversed-phase analytical column (2.0 × 100 mm, 3-μ particles) (Waters). Gradient elution was done at a flow rate of 0.3 ml/min. Phase A was 0.1% formic acid in water (v/v) and phase B was acetonitrile. The column oven and auto-sampler temperatures were set to 40 ± 3°C and 5 ± 3°C, respectively. The injection volume of the sample extract was 5 μl. Positive electrospray ionization was used for the detection of folate metabolites. The protonated molecular ions [M + H]^+^ of the folates were selected as the precursor ions. One specific mass transition (m/z) was chosen for each compound using the multiple reaction monitoring mode function of the instrument for quantitation. The optimized source-dependent parameters, which included capillary voltage, desolvation temperature, desolvation gas flow, cone gas flow, and collision gas flow, were maintained constantly at 2.92 kV, 50°C, 600 l/h, 150 l/h, and 0.15 ml/min, respectively, throughout the analysis. Detailed analytical conditions have been previously described[34]. We used Mass Lynx 4.1 software for instrument control and data acquisition.

### Extraction and Quantification of NADPH

Cells were harvested in the mid-logarithmic phase of growth and quenched by liquid nitrogen. The concentrations of NADPH in the *L. lactis* strains were measured using the NADP/NADPH Quantification Kit (Sigma-Aldrich, USA, MAK038) following the instructions of the supplier. The values of optical density at 600 nm (OD_600_) were converted to dry cell weight according to the following equation: 1 OD_600_ = 0.31 g/l.

## Results and Discussion

### Effects of Overexpression of 5-MTHF Pathway Genes on 5-MTHF Production

**Fig. 2:**
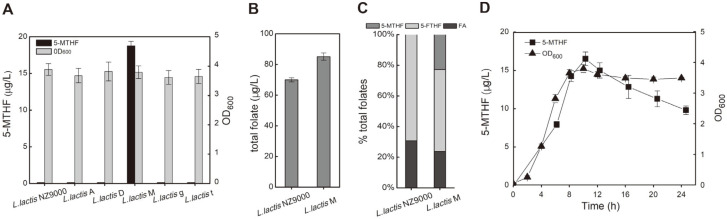
Effects of overexpression of 5-MTHF pathway genes on 5-MTHF production. (**A**) Effects of overexpression of 5-MTHF pathway genes on 5-MTHF production and cell growth. (**B**) Comparison of total folates (folate, 5-MTHF, and 5-FTHF) concentrations of *L. lactis* NZ9000 and *L. lactis* M. (**C**) The ratio of folate, 5-MTHF, and 5-FTHF in total folates. (**D**) Time course of MTHF production and cell growth of *L. lactis* M.

Microbial synthesis of folate has been reported, but the production of 5-MTHF by microbial fermentation is unclear [21-23]. *L. lactis* NZ9000 harbors the complete native folate and 5-MTHF synthetic pathways. However, 5-MTHF cannot quantitatively accumulate in cells due to tight regulation of the 5-MTHF pathway. Therefore, to strengthen the production of 5-MTHF to permit 5-MTHF accumulation, the genes of the 5-MTHF synthesis pathway need to be overexpressed. To achieve this, first, *metF* encoding MTHFR was overexpressed in *L. lactis*, which led to the accumulation of intracellular 5-MTHF. The intracellular level reached 18 μg/l (Fig. 2A). Next, the genes encoding DTHR (*dfrA*), thymidylate synthase (*thyA*), glycine hydroxymethyltransferase (*glyA*), and methylenetetrahydrofolate dehydrogenase (*folD*) were also overexpressed in *L. lactis* NZ9000. However, there was no significant improvement in 5-MTHF production in these recombinant strains compared to the initial host (Fig. 2A). Moreover, overexpression of MTHFR in *L. lactis* increased production of the total folates from 70 μg/l to 83 μg/l, representing an increase of 23% (Fig. 2B). The prevalence of 5-MTHF in total folates increased from 0 to 20%, while 5-FTHF accounted for the largest proportion of folates in *L. lactis* M reduced to 53% (Fig. 2C). In addition, we found that the synthesis of 5-MTHF appeared to be closely coupled to cell growth, as 5-MTHF was rapidly synthesized and accumulated during the logarithmic growth phase, and the titer gradually decreased during stationary phase (Fig. 2D).

These results indicated that MTHFR, which catalyzes the reduction of 5,10-methylenetetrahydrofolate in the formation of 5-MTHF, is a key rate-limiting enzyme in the synthesis of 5-MTHF. Compared to the strain engineered to overexpress *metF*, the low expression level of the *metF* gene in the wild-type strain may be one of the reasons for limited 5-MTHF accumulation. The enhanced production of MTHFR was demonstrated to be necessary for 5-MTHF overproduction. Moreover, 5-MTHF is an important methyl donor and participates in amino acid synthesis, DNA methylation, and other metabolic processes that are closely related to cell growth [35]. During the logarithmic growth phase, rapid cell proliferation increases the need for a carbon source for cellular metabolism and nucleotide biosynthesis, and 5-MTHF is rapidly synthesized to meet the needs of cell growth [36]. When the cells enter stationary phase, cell proliferation slows with a reduced 5-MTHF synthesis rate, resulting in gradual consumption of 5-MTHF.

### Effects of Combinatorial Overexpression of Genes in 5-MTHF Synthesis Pathway on 5-MTHF Production

**Fig. 3:**
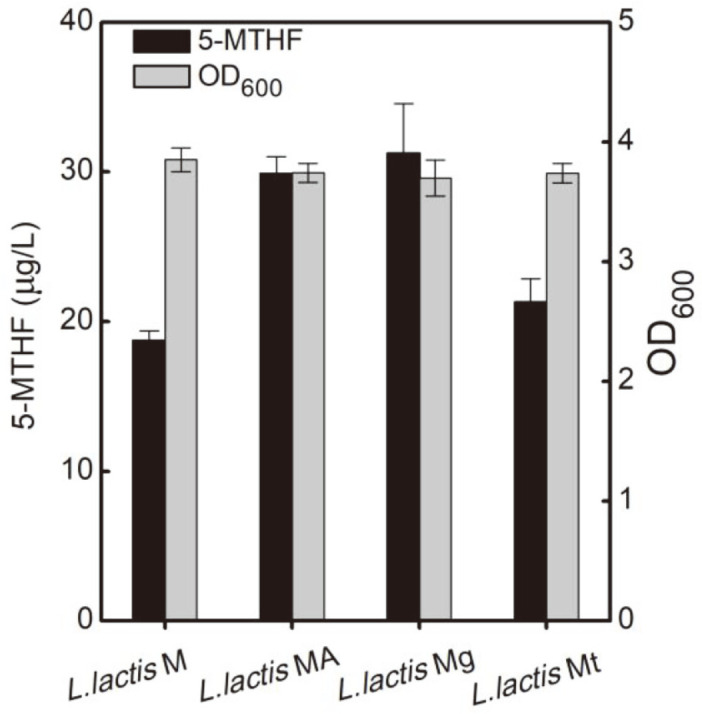
Effects of co-overexpression of 5-MTHF synthesis pathway genes in combination with *metF* gene overexpression on 5-MTHF production and cell growth.

Various folates can be mutually converted in vivo by different 5-MTHF biosynthesis pathways (Fig. 1). Complex metabolic pathways and reversible conversion processes could be the potential reasons for limiting 5-MTHF synthesis. To further increase 5-MTHF production, the genes in the 5-MTHF synthetic pathway, including *drfA* (encoding DHTR), *glyA* (encoding glycine hydroxymethyltransferase), and *thyA* (encoding thymidylate synthase) were co-overexpressed in combination with *metF* (encoding MTHFR). Further overexpression of *dfrA* and *glyA* on the basis of the overexpression of *metF*, to obtain the *L. lactis* MA and *L. lactic* Mg recombinant strains, led to the 59% and 67% increase in the 5-MTHF titer, respectively. However, cooverexpression of the *thyA* and *metF* genes did not significantly promote 5-MTHF synthesis (Fig. 3).

DHF is reduced to THF by DHFR with the participation of NADPH, and folate can only be methylated after a step-wise conversion to reduced folate and then to 5-MTHF [37]. Overexpression of DHFR allows folate and DHF to be converted more rapidly to THF, which was beneficial to promote the folate methylation process and promoted the synthesis of 5-MTHF. In addition, THF can be converted to 5,10-methylenetetrahydrofolate by a one-step metabolic reaction catalyzed by glycine hydroxymethyltransferase using serine as a methyl donor [38]. The conversion of THF to 5-MTHF in this pathway reduced the reversible interconversion between different folates. The findings indicated that reducing the complex reversible interconversion between folate derivatives and shortening the metabolic pathways are effective in accelerating the conversion of folate to 5-MTHF.

### Strengthening the Supply of the Key Precursor, Folate, by Overexpression of the Key Folate Synthesis Gene, *folE*

**Fig. 4:**
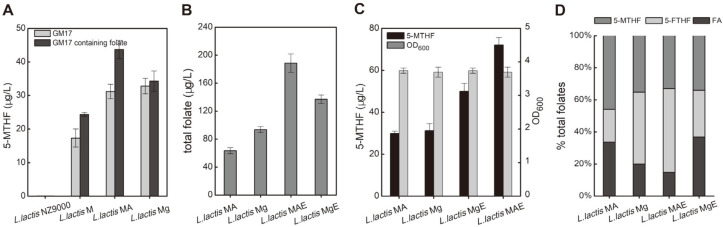
Effect of strengthening key precursor folate supply on 5-MTHF synthesis and cell growth. (**A**) Effects of folate addition into culture medium on 5-MTHF production. (**B**) Effects of overexpression of *folE* gene on total folates (folate, 5-MTHF, and 5-FTHF) production. (**C**) Effects of overexpression of *folE* gene on 5-MTHF production. (**D**) The ratio of folate, 5-MTHF, and 5-FTHF in total folates.

Since folate was the key precursor for 5-MTHF synthesis, increasing the folate supply may be an effective approach to promote 5-MTHF synthesis. To test whether folate supply is the limiting factor for 5-MTHF production, the effect of folate addition to the medium on 5-MTHF production was investigated. Enhanced supply of folate promoted the synthesis of 5-MTHF, especially for strains that overexpressed DHTR (Fig. 4A). Thus, increasing the native folate synthesis of *L. lactic* was a promising way to promote 5-MTHF synthesis. The *folE* gene is one of the key genes in folate biosynthesis, and overexpression of the *folE* gene reportedly increased total folate by more than two times in *L. lactis* NZ9000[21]. Presently, the overexpression of the *folE* gene in *L. lactis* MAE and *L. lactis* MgE increased the total folate concentration, which reached 188 μg/l and 137 μg/l, respectively. Compared with strains *L. lactis* MA and *L. lactis* Mg, which did not feature overexpression of the *folE* gene, total folates increased 3.0-fold and 1.5-fold, respectively, with improved 5-MTHF reaching 72.1 μg/l and 52.7 μg/l in *L. lactis* MAE and *L. lactis* MgE, respectively (Figs. 4B and 4C). Compared with strains *L. lactis* MA and *L. lactis* Mg, 5-MTHF production was enhanced by 135% and 67% in *L. lactis* MAE and *L. lactis* MgE, respectively. In *L. lactis* MAE, overexpression of the *folE* gene resulted in a significant increase in 5-MTHF production, while the proportion of 5-MTHF in total folate did not increase, but rather decreased from 46% to 32%, and the 5-FTHF ratio increased significantly from 20% to 52% (Fig. 4D). Further engineering will be needed to improve 5-MTHF production.

The finding that the addition of folate in fermentation medium promoted the synthesis of 5-MTHF indicated that the supply of folate was a limiting factor of 5-MTHF synthesis. Thus, increased folate synthesis by the strain is necessary to improve 5-MTHF production. Microbes synthesize folate *de novo* with pterin, *p*ABA, and glutamic acid, and pterin synthesis is pivotal in folate biosynthesis. The biosynthesis of pterin begins with GTP. This reaction is also the rate-limiting reaction of folate synthesis [39]. GTP cyclohydrolase I and GTP cyclohydrolase II control two competing pathways in GTP flux in the folate synthesis pathway and the riboflavin synthesis pathway [21, 23]. Presently, the overexpression of GTP cyclohydrolase I caused a greater flux of GTP to the folate synthesis pathway, resulting in a significant increase in folate yield, which also increased the production of 5-MTHF.

### Increasing Intracellular NADPH Levels Improves 5-MTHF Production

**Fig. 5:**
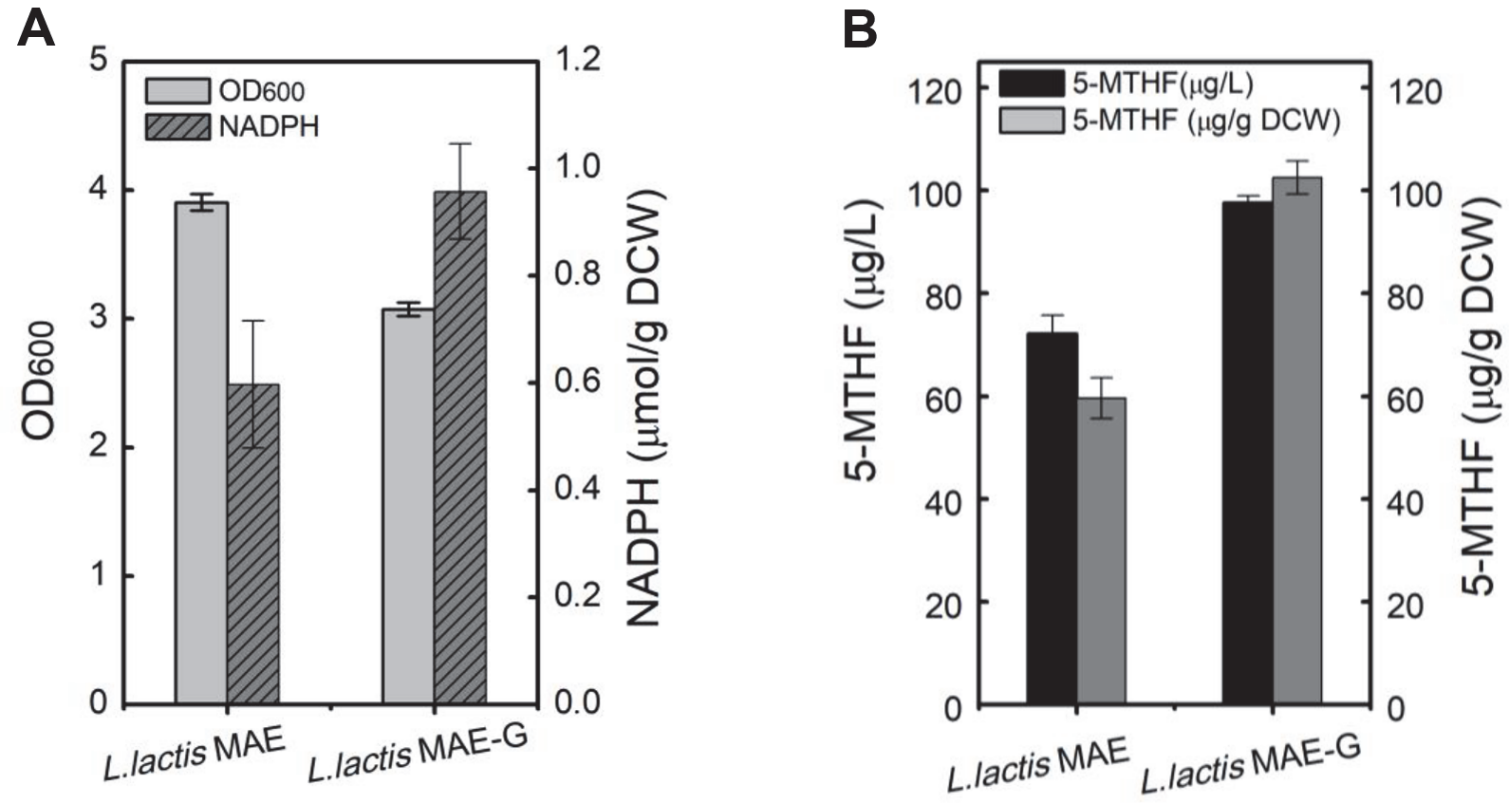
Increasing intracellular NADPH levels for improving 5-MTHF production. (**A**) Effect of overexpression of the G6PDH gene on intracellular NADPH concentrations and cell growth. (**B**) Effect of overexpression of the G6PDH gene on 5-MTHF production.

In the 5-MTHF synthesis pathway, producing 1 mol of 5-MTHF consumed 4 mol of NADPH using folate as substrate (Fig. 1). Therefore, efficient NADPH supply and regeneration are important for 5-MTHF synthesis. To increase the content of intracellular NADPH, G6PDH was overexpressed to strengthen the pentose phosphate pathway for NADPH generation. G6PDH overexpression facilitated NADPH supply and increased the intracellular NADPH concentration by 60% (Fig. 5A), which led to the 35% increase in 5-MTHF production, reaching 97 μg/l in *L. lactis* MAE-G (Fig. 5B).

Most of the biosynthetic pathways for secondary metabolites are influenced by carbon metabolism and supply of cytosolic NADPH [40]. NADPH is mainly synthesized by a central carbon metabolic pathway, which can be strengthened by redistributing the glycolysis flux to the pentose phosphate pathway [41]. Overexpressing G6PDH strengthens the pentose phosphate pathway, which accelerates the rate of NADPH reduction, thereby increasing intracellular NADPH content [32, 41]. During the synthesis of 5-MTHF, multi-step reactions that include the conversion of folate to THF and the reduction of methylene require the participation of NADPH. Therefore, maintaining a higher level of NADPH in cells is beneficial to promote the synthesis of 5-MTHF. At the same time, NADPH is vital for the oxidative defense mechanism of cells, since it is able to maintain glutathione in a reduced state and counteract reactive oxygen species(ROS) [41, 42], which can prevent folates from being oxidized and maintain 5-MTHF stability.

### Adjusting the Ratio of Intracellular Folate Derivatives Improves 5-MTHF Production

**Fig. 6:**
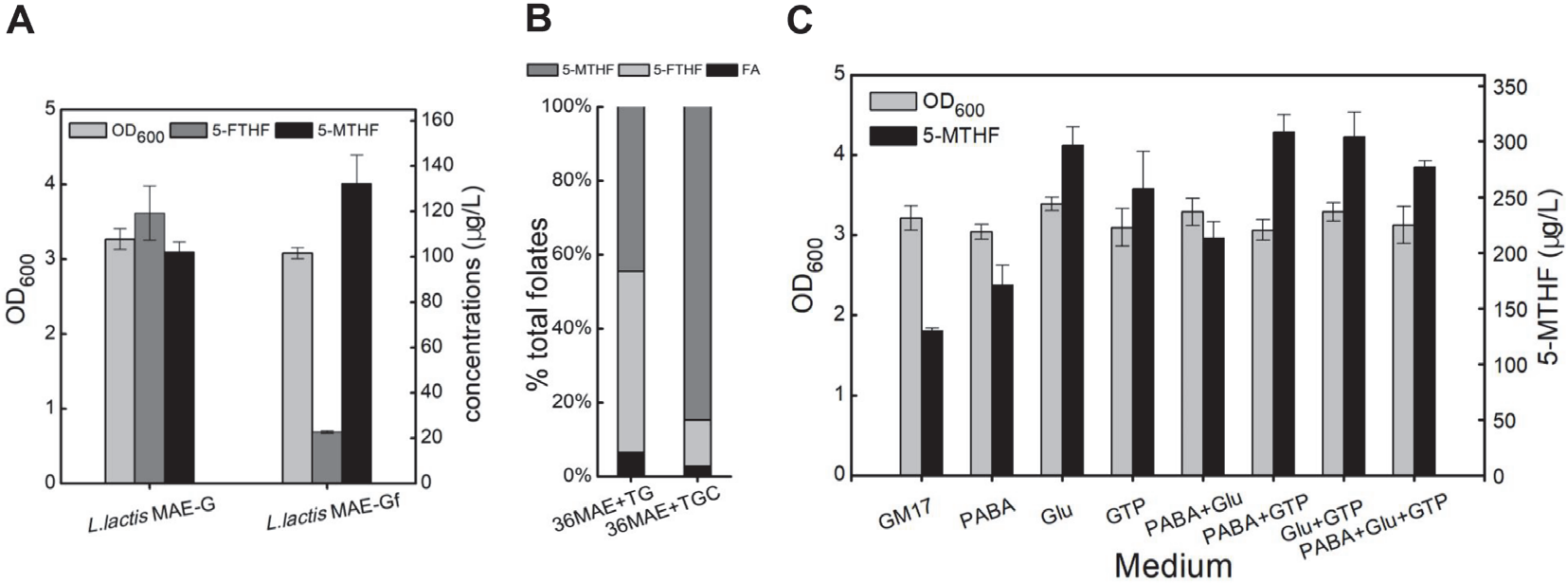
Effects of overexpression of 5-formyltetrahydrofolate cyclo-ligase and adding key folate precursors on 5-MTHF production. (**A**) Effects of overexpression of *fau* gene encoding 5-formyltetrahydrofolate cyclo-ligase on 5- MTHF production, 5-FTHF production and cell growth. (**B**) The ratio of folate, 5-MTHF, and 5-FTHF in total folates. (**C**) Effects of adding precursor of folate in medium on 5-MTHF production. 50 mg/l *p*ABA, 300 mg/l glutamate, and 30 mg/l GTP were respectively or combinatorially added into the fermentation medium.

The finding that 5-FTHF accounted for a large proportion of total folates in *L. lactis* is similar to results from fungi [23]. To further increase the yield of 5-MTHF, the formation of other folates derivatives should be reduced. The 5-formyltetrahydrofolate cyclo-ligase catalyzes the conversion of 5-FTHF to 5,10-methenyltetrahydrofolate, which facilitates folate metabolic flux back to the 5-MTHF synthesis pathway and reduces the formation of the 5-FTHF by-product. The overexpression of 5-formyltetrahydrofolate cyclo-ligase resulted in substantial decrease in the titer of 5-FTHF from 119 μg/l to 23 μg/l, and the 32% increase in 5-MTHF from 100 μg/l to 132 μg/l without significant effects on cell growth (Fig. 6A). The ratio of 5-FTHF was also sharply reduced from 48% to 12% (Fig. 6B). To further boost 5-MTHF biosynthesis, the effects of adding key precursors of folate, including GTP, *p*ABA, and glutamic acid, on 5-MTHF production were studied. The addition of *p*ABA and GTP enhanced the 5-MTHF titer by 2.3 times, reaching 300 μg/L (Fig. 6C).

Biosynthesized folates contain a variety of folate derivatives, with 5-FTHF accounting for a large proportion [43]. Therefore, reducing or blocking the formation of 5-FTHF is necessary to increase the production of 5-MTHF. However, the synthetic pathway of 5-FTHF has not been annotated in *L. lactis*. Presently, the strategy of converting 5-FTHF to other folate derivatives in 5-MTHF synthesis pathway was explored. Overexpression of 5-formyltetrahydrofolate cyclo-ligase converted 5-FMTH to 5,10-methenyltetrahydrofolate, which reduced 5-FMTH formation and increased 5-MTHF synthesis. In addition, the NADPH synthesis pathway was strengthened [44], which should be sufficient for NADPH supply and to further promote the metabolism of 5,10-methenyltetrahydrofolate to 5-MTHF. The folate molecule is composed of pterin, *p*ABA, and glutamic acid residues. These precursors were directly added to the medium, which markedly boosted folate synthesis, which was beneficial and important in promoting 5-MTHF synthesis.

The production of folates in *L. lactic* is substantially lower than the production by *Ashbya gossypii* (6,595 μg/l total folates with 1,253 μg/l 5-MTHF) [23]. However, there are some potential advantages in using *L. lactic* for 5-MTHF production. These include short fermentation period and GRAS status. Moreover, this research focused on increasing the yield of a particular reduced form of folate, 5-MTHF, which has potential advantages compared to non-reduced forms of folates such as nutraceuticals and pharmaceuticals. The proportion of 5-MTHF in total folates that was presently achieved is higher than previously reported [23]. In order to further increase the yield of 5-MTHF, two strategies will be explored. First, competition pathways involved in folate synthesis will be inhibited, which will allow more metabolic flux to the folate synthesis pathway. Second, the 5-MTHF consumption pathway will be identified and repressed.

In conclusion, the biosynthesis of 5-MTHF by *L. lactis* was improved by systematic pathway engineering, including: 1) identifying and overexpressing the key rate-limiting enzyme MTHFR, 2) strengthening 5-MTHF biosynthesis and folate supply by co-expression of the *drfA*, *metF*, and *folE* genes, 3) increasing NADPH supply by overexpression of G6PDH, and 4) increasing the proportion of 5-MTHF in various folates by overexpressing 5-formyltetrahydrofolate cyclo-ligase. Finally, the 5-MTHF titer reached 300 μg/L with key precursor addition using the engineered *L. lactis* strain. We hope the developed engineering strategies and 5-MTHF-producing strain will enable efficient 5-MTHF bioproduction in *L. lactis*.

## Supplemental Material

Supplementary data for this paper are available on-line only at http://jmb.or.kr.

## Figures and Tables

**Table 1:** Strains and plasmids used in this study.

Name	Relevant characteristics	Reference
Strains		
*L. lactis* NZ9000	*L. lactis subsp. cremoris,* plasmid free	[45]
*L. lactis* A	*L. lactis* contains plasmid pMG36e-A	This work
*L. lactis* D	*L. lactis* contains plasmid pMG36e-D	This work
*L. lactis* M	*L. lactis* contains plasmid pMG36e-M	This work
*L. lactis* t	*L. lactis* contains plasmid pMG36e-t	This work
*L. lactis* g	*L. lactis* contains plasmid pMG36e-g	This work
*L. lactis* MA	*L. lactis* contains plasmid pMG36e-MA	This work
*L. lactis* Mg	*L. lactis* contains plasmid pMG36e-Mg	This work
*L. lactis* Mt	*L. lactis* contains plasmid pMG36e-Mt	This work
*L. lactis* MAE	*L. lactis* contains plasmid pMG36e-MAE	This work
*L. lactis* MgE	*L. lactis* contains plasmid pMG36e-MgE	This work
*L. lactis* MAE-G	*L. lactis* contains plasmid pMG36e-MAE and pTD6-G	This work
*L. lactis* MAE-Gf	*L. lactis* contains plasmid pMG36e-MAE and pTD6-Gf	This work
Plasmids		
pMG36e	Em^r^; expression vector carrying P_32_ promoter	[46]
pTD6	Tet^r^; expression vector	[47]
pMG36e-A	Em^r^; pMG36e derivative containing *drfA* gene	This work
pMG36e-D	Em^r^; pMG36e derivative containing *folD* gene	This work
pMG36e-M	Em^r^; pMG36e derivative containing *metF* gene	This work
pMG36e-t	Em^r^; pMG36e derivative containing a *thyA* gene	This work
pMG36e-g	Em^r^; pMG36e derivative containing a *glyA* gene	This work
pMG36e-MA	Em^r^; pMG36e-M derivative containing *metF* and *drfA* gene	This work
pMG36e-Mg	Em^r^; pMG36e-M derivative containing *metF* and *glyA* gene	This work
pMG36e-Mt	Em^r^; pMG36e-M derivative containing *metF* and *thyA* gene	This work
pMG36e-MAE	Em^r^; pMG36e-MA derivative containing *metF*, *drfA* and *folE* gene	This work
pMG36e-MgE	Em^r^; pMG36e-Mg derivative containing *metF*, *glyA* and *folE* gene	This work
pTD6-G	Tet^r^; pTD6 derivative containing G6PDH gene	This work
pTD6-Gf	Tet^r^; pTD6 derivative containing G6PDH and *fau* gene	This work
