# Supplementary material for: Engineering of Biosynthesis Pathway and NADPH Supply for Improved L-5-Methyltetrahydrofolate Production by Lactococcus lactis
Source: J Microbiol Biotechnol. 2019 Dec 24;31(1):154–62. doi: 10.4014/jmb.1910.10069 (PMC9705839; doi:10.4014/jmb.1910.10069)
Supplement: Supplementary file 1 [file jmb-31-1-154-supple.pdf]

J. Microbiol. Biotechnol.
https://doi.org/10.4014/jmb.1910.10069

Table S1 Primers used in this work

Name	Sequence
pMG36e-F	TGACCGGTAAAATTTAATATTTTGAACCTTGCTT
pMG36e-R	TTCAAAATTCCTCCGAATATTTTTTTACCTACCTAGT
metF-F	ATATTCGGAGGAATTTTGAAATGACAAGTGATTCTAAAATTCTATCTTTTGAAGTTTTTCC
metF-R	TATTAAATTTTACCGGTCATTTATGTATTTATTTTAAATAAAGATGAGATTGAAGAATGAATGGAACGTG
drfA-F	ATATTCGGAGGAATTTTGAAATGATAATTGGAATATGGGCAGAAGATGAG
drfA-R	TATTAAATTTTACCGGTCATTCATGGTTGTTTCACTTTTTCATATTTTTTAATCGTAAAAG
folD-F	ATATTCGGAGGAATTTTGAATTGAAAGTCGGCTTATGGTATAATAGTTTTATGAACT
folD-R	TATTAAATTTTACCGGTCATTCAGTTACTATTTTCATTCATTTTTCTTGTTGCCG
thyA-F	ATATTCGGAGGAATTTTGAAATGACTTACGCAGATCAAGTTTTTAAACAAAATATCC
thyA-R	TATTAAATTTTACCGGTCATTAAATTGCTAAATCAAATTTCAATTGAGGTTTTACTGGC
glyA-F	ATATTCGGAGGAATTTTGAAATGATTTTTGATAAAGAAGATTTTGAAAGCTTTGACC
glyA-R	TATTAAATTTTACCGGTCATTTATAATGGAAATTGATGTGTTAACTCTAAGGCAGAT
drfA-metF-F	ATATTCGGAGGAATTTTGAAATGATAATTGGAATATGGGCAGAAGATGAG
glyA-metF-F	ATATTCGGAGGAATTTTGAAATGATTTTTGATAAAGAAGATTTTGAAAGCTTTGACC
thyA-metF-F	ATATTCGGAGGAATTTTGAAATGACTTACGCAGATCAAGTTTTTAAACAAAATATCC
metF-R2	TTCAAAATTCCTCCGAATATTTATGTATTTATTTTAAATAAAGATGAGATTGAAGAATGAATGGAACG
folE-F	ATATTCGGAGGAATTTTGAAATGCAAACAACTTATTTAAGCATGGGAAGTAAT
folE-R	ATATTAAATTTTACCGGTCATCATAAAAGACTTTCTAAAAATTCCATTCTTTTTTCGCC
drfA-folE-R	TTCAAAATTCCTCCGAATATTCATGGTTGTTTCACTTTTTCATATTTTTTAATCGT
glyA-folE-R	TTCAAAATTCCTCCGAATATTTATAATGGAAATTGATGTGTTAACTCTAAGGCAGAT
pTD6-F	GCAAAGAATGGCGGAAACGTAAAAG
pTD6-R	GGTCGACAAGCTTCTGCAGATCT
G6PDH-F	CAGAAGCTTGTCGACCCTTATAAGATTAATGCTTCACAAGAGTTTTTGAGGC
G6PDH-R	TTCCGCCATTCTTTGCAAAATTAAAAGGAATTTTACCTGTGGAAAAATAAATCC
fua-F	ATATTCGGAGGAATTTTGAAATGGAAAGTAAAGTAGAAAATCGCAAAAAAAATATTACAAATG
fua-R	TAAATTTTACCGGTCACTGGTTAAAATTCATTATTTTTTCTCCCTATAAAAAGTTTTTTTACTGCAATATC
